# Detector Development for the abBA Experiment

**DOI:** 10.6028/jres.110.065

**Published:** 2005-08-01

**Authors:** P.-N. Seo, J. D. Bowman, G. S. Mitchell, S. I. Penttila, W. S. Wilburn

**Affiliations:** Los Alamos National Laboratory, Los Alamos, NM 87545, USA

**Keywords:** dead layer, electron-backscattering event, neutron beta decay, silicon detector

## Abstract

We have developed a new type of field-expansion spectrometer to measure the neutron beta decay correlations (a, b, B, and A). A precision measurement of these correlations places stringent requirements on charged particle detectors. The design employs large area segmented silicon detectors to detect both protons and electrons in coincidence. Other requirements include good energy resolution (< 5 keV), a thin dead layer to allow observation of 30-keV protons, fast timing resolution (~1 ns) to reconstruct electron-backscattering events, and nearly unity efficiency. We report results of testing commercially available surface-barrier silicon detectors for energy resolution and timing performance, and measurement of the dead-layer thickness of ion-implanted silicon detectors with a 3.2 MeV alpha source.

## 1. Introduction

Precision measurements of neutron beta decay correlations, *a*, *b*, *B*, and *A*, provide important tests of the electroweak standard model: a test of unitarity of the first row of the CKM matrix, a test of the CVC hypothesis, and a sensitive search for right-handed currents or scalar or tensor bosons.

The abBA collaboration has developed a conceptual design for a new type of field-expansion magnetic spectrometer to measure neutron decay correlation coefficients, *a*, *b*, *B*, and *A*, with a polarized pulsed cold neutron beam [1,2]. abBA aims to measure the correlations to an order of magnitude better accuracy than previous measurements, where the accuracy was limited by systematic errors caused by 1) neutron polarimetry, 2) poor detector properties such as resolution, efficiency, stability, and homogeneity, and 3) large backgrounds. To improve systematic uncertainties, abBA will utilize the pulsed nature of the neutron beam at SNS, which allows an accurate beam polarization measurement by a time-of-flight technique for *A* and *B* [3]. Coincidences between electrons and protons from the neutron decay will be used to suppress backgrounds.

The decay rate for polarized neutrons as a function of electron energy (*E*_e_) and neutron life time (*τ*_n_) is given by [4],

$$dW\propto \frac{1}{\tau_{n}}F(E_{e})\left[1+a\frac{P_{e}\cdot P_{\nu }}{E_{e}E_{\nu }}+b\frac{m_{e}}{E_{e}}+B\frac{\sigma_{n}\cdot P_{\nu }}{E_{\nu }}+A\frac{\sigma_{n}\cdot P_{e}}{E_{e}}\right],$$ (1)

where ***P***_e_ and ***P***_ν_ are the outgoing electron and neutrino momenta, ***σ***_n_ is the neutron spin, *E*_ν_ is the neutrino energy, and *F*(*E*_e_) is electron energy spectrum. To measure the decay parameters both electrons (50 keV to 780 keV) and protons (< 30 keV) must be detected.

The charged decay particles, electrons and protons, are guided through a magnetic field into two large area and 2π solid angle Si detectors mounted on both ends of the field-expansion spectrometer. The protons are accelerated by an electric field up to 30 keV before they reach the detectors. The dead layer of the Si detector has to be thin enough (≈ 100 nm) to allow a low-energy proton detection with energy resolution better than 5 keV. Measurement of the electron energy depends only on the characteristics of the detectors. Electrons drift into the detectors following the magnetic field in their spiral path. However, about 20 % of them backscatter. Most of these electrons drift back to the original detector because of the magnetic field pinch, but a small fraction have enough kinetic energy to cross the spectrometer and hit the opposite detector. The timing of electron hits must be resolved sufficiently to determine which detector was hit first in order to reconstruct events for *a* [2] and *A*. The required timing resolution is about 1 ns.

The size of the detector is defined by the magnetic field expansion for charged particles. The conceptual design of the Si detector has 15 cm diameter and 2 mm thickness and consists of 100 active pixels. Each pixel has a hexagonal shape with a 1 cm^2^ area. To verify that the conceptual detector meets design criteria, we chose commercially available surface-barrier Si detectors to measure timing resolution and ion-implanted Si detectors to measure available dead layers.

## 2. Timing Measurement

In the abBA experiment, some backscattered electrons will deposit their energy in both detectors. In such cases, in order to reconstruct the events, good timing resolution is required to distinguish which detector the electrons hit first. Using a ^90^Sr beta source with maximum energy of 2.28 MeV, timing resolution was measured for surface-barrier Si detectors using a fast plastic scintillator as a time reference.

**Fig. 1:**
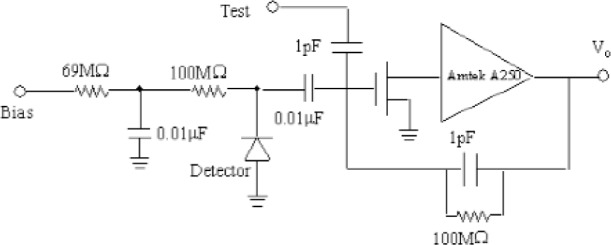
Preamplifier circuit with a FET for timing measurements with a Si detector.

In the measurement, the electrons from the source travel through the 2.8 mm thick plastic scintillator producing fast signals that are used as a timing reference. They then enter a vacuum chamber through a thin Al window, and finally hit a 1 cm^2^ area and 2 mm thick Si detector [5], which was cooled close to LN_2_ temperature. The Si detector was biased with 160 V. Electrons interacting with silicon create electron-hole pairs. Due to the applied electric field, electrons move towards the anode and holes move towards the cathode to generate electrical signals, which were amplified with the circuit shown in Fig. 1.

**Fig. 2:**
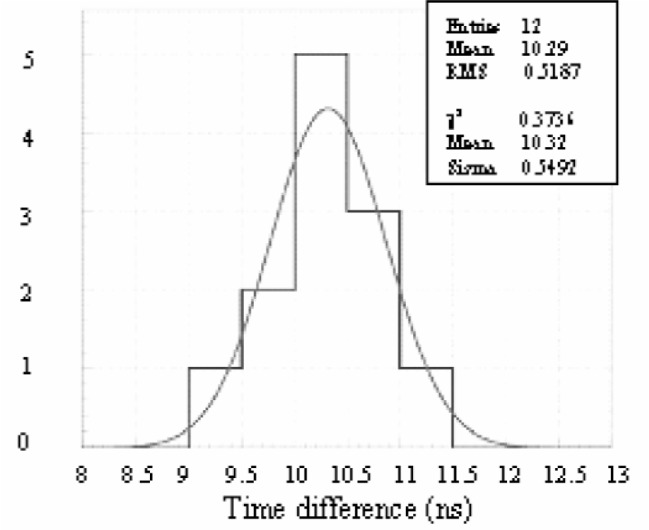
A histogram of 12 timing measurements with a surface-barrier Si detector.

Si-detector events with a scintillator coincidence were recorded with an oscilloscope. In order to determine the time of the signal with the respect to the scintillator, a linear fit was used to determine slopes in the preamp signal and in the discriminator output of the scintillator. The time difference between the signals was obtained by comparing the times to reach half of maximum signal amplitude. The time difference between the two signals ranged from 9.02 ns to 11.06 ns with standard deviation of 0.59 ns for 12 waveforms from events with energy deposition of 170 keV as shown by the histogram in Fig. 2. The experimental FWHM is 2.35*σ* = 1.4 ns, with uncertainty of 0.5 ns. The expected FWHM can be calculated using charge collection time, a FET noise, and a signal amplitude and capacitance, using the following formula,

$$\Gamma =2.35\frac{V_{n}C\delta E}{E}\sqrt{\frac{T_{\text{rise}}}{2}},$$

where *V*_n_ is the FET noise, ≈ 10^−9^ [V/sqrt(Hz)], C = 20 pF, *δ*E is the energy per electron-hole pair at room temperature, 3.62 eV/pair, and *T*_rise_ is measured rise time, 55.4 ns. The expected value, *Γ* = 1.02 ns, agrees with measured time resolution. We expect that timing resolution would be improved by increasing the detector bias to increase drift velocities of both electron and hole in the silicon and by cooling the FET to reduce electronic noise [6].

With 59.5 keV gamma rays from a ^241^Am source, we measured 8.1 keV energy resolution with an FET at room temperature and energy resolution was improved to 3.6 keV with the cooled FET.

## 3. Measurement of Detector Dead Layer Thickness

The dead layer on the surface of a detector serves to protect the Si surface. The detector dead layer is a consequence from the fabrication method, which starts with a homogeneous crystal of *p*-type materials and then converts a region of the crystal near the surface from a *p*-type to an *n*-type material. This creates a junction at some distance from the surface, typically 100 nm to 200 nm. A particle interacting with this layer will lose its energy without detection and therefore it is important to minimize the thickness of the dead layer. This is one of the main issues in detector development for the abBA experiment where the dead layer must be thin enough to detect 30 keV protons.

We have demonstrated the capability to measure thin dead layers with four ion-implanted sample detectors and each segmented into four 1 cm^2^ active areas and having ≈ 300 µm depletion depth [7]. Two of the detectors are not coated and two are coated with gold on the surface.

**Fig. 3:**
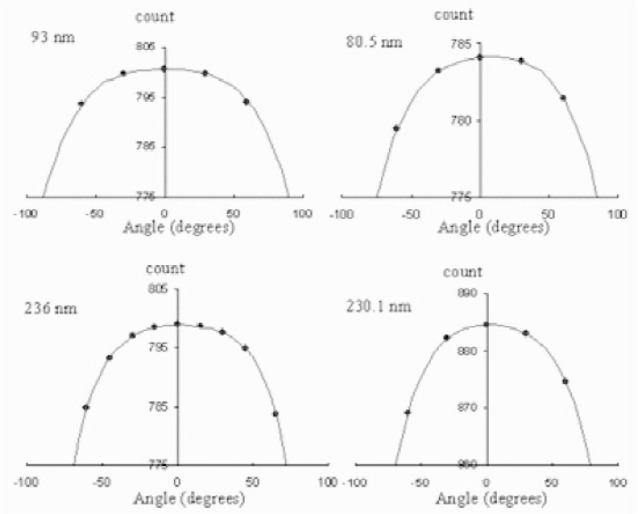
The results of dead layer measurement for four ion-implanted silicon detectors are fitted (solid line). Detectors were rotated up to 60°.

The detector dead layers were determined by measuring the energy loss of 3.2 MeV *α*-particles from a radioactive ^148^Gd source and by using a calculated d*E*/d*x* [8]. The detectors were mounted on a rotating stage in a vacuum chamber and spectra were taken for several angles. The measured *α*-particle energy was fitted to *E* = *E*_0_ − *ΔE*/cos*θ*, where *E*_0_ is the incident particle energy, *ΔE* is the energy loss in the detector dead layer, and *θ* the angle of the detector, from nominal incidence. The results of the dead layer measurements are shown in Fig. 3. We obtained 93 nm, 80.5 nm, 236 nm, and 230.1 nm for the non-coated detectors and coated detectors respectively. Detectors coated with gold have twice thicker dead layers than detectors without a gold coating.

## 4. Conclusion

Detector development for the abBA experiment has made significant progress. We measured the timing resolution with commercially available surface-barrier Si detectors. Measured timing resolution of 1.4 ns agrees with the expected timing resolution of 1.02 ns within 30 % uncertainty. The dead layers of commercially available ion-implanted Si detectors were measured and 100 nm dead layers are sufficiently thin to allow detection of 30 keV protons. Further development works such as dead layer measurement with 30 keV protons, improvement of timing resolution remain before a large area, segmented, and thin dead-layer detector can be produced.
